# Pension level, subjective wellbeing, and preference of care model among elderly people: An empirical study based on structural equation modeling

**DOI:** 10.3389/fpubh.2023.1104556

**Published:** 2023-02-09

**Authors:** Chenyang Shao, Wenshun Li

**Affiliations:** ^1^Business School, Beijing Normal University, Beijing, China; ^2^School of Economics and Management, Tongji University, Shanghai, China

**Keywords:** pension, subjective wellbeing, elderly care model, structural equation model, aging

## Abstract

The problem of population aging is becoming increasingly serious, and the elderly care model has become the social focus in China. It is urgent to improve the traditional home based elderly care model and increase the recognition of the residents for the socialized elderly care model. Based on the data of the 2018 China Longitudinal Aging Social Survey (CLASS), this paper uses structural equation model (SEM) to empirically test the impact of the elderly group's social pension level and subjective well–being on their choice of various care models. The results show that the improvement of elderly's pension level can significantly inhibit their choice of home–based care model, and promote their choice of community and institution care model. Subjective well–being can play a mediating role in the home–based and community care model choices, but the indirect approach mediated by subjective well–being only plays a supplementary role. In addition, the heterogeneity analysis shows that there are discrepancies in the impact and path for the elderly with different gender, age, household registration, marital status, health status, education level, number of children, and gender of children. The results of this study will help the improvement and development of social pension policy, optimizing the structure of residents' elderly care model and the process of “active aging”.

## 1. Introduction

Alongside economic and social development in China, the aging population is becoming increasingly notable. Data from the seventh national population census, conducted in 2020, indicate that China's population is in a state of structural imbalance, with a reduction in the size of the working-age population and in the number of women of childbearing age. Total fertility rate and number of births are declining, while the proportion of elderly people is rising. At the same time, among the growing number of elderly people, a significant portion are facing significant health risks due to insufficient security of medical care, making care of the elderly a social concern (1–6). In recent years, reform of the elderly care system has been promoted in China; essentially, the country has developed a multi-level service system of elderly care, with “home-based care as the foundation, community as the support, and institutions as the supplement” (7–11). Whether the choice is based on physical or psychological considerations, choosing a suitable elderly care model is crucial to improve quality of life among elderly people. In China, due to traditional beliefs and other psychological factors, elderly people prefer to spend their old age at home and have certain prejudices toward and concerns about elderly care centers or nursing homes. Therefore, elderly people are usually more willing to choose home-based care than to opt for care in the community or institutional care (12–15). As the number of children decreases and the size of Chinese families shrinks, the home-based care model is becoming increasingly inadequate to cope with the heavy pressure caused by the changing structure of the population in terms of age. Therefore, it is necessary to reform the traditional elderly care model and increase recognition of the community and institutional models for elderly care. Such changes will promote the provision of public and social elderly care and the high-quality development of the entire elderly care system in China.

Studies have shown that the choices of elderly care model made by elderly people are influenced by a variety of factors, such as personal characteristics, family structure, care provided by children, income, and degree of social security coverage (16–18). Based on international findings, any public pension policy may impact citizens' individual pension arrangements (19–21). Therefore, the implementation of China's social endowment insurance policy and the receipt of pension provide new possibilities for transforming the inherent traditional care model of the elderly in China and rapidly entering the process of socialized. Therefore, it is assumed that provision of an appropriate pension can help elderly people to become resilient to unpredictable risks and can improve their quality of life, especially *via* the provision of a subsidy to their families. Elderly individuals' dignity, sense of access, satisfaction, and security can be enhanced efficiently through improvements in their pension level. Community- and institution-based elderly care will be more acceptable to elderly people if they have a decent pension; thus, promoting a shift from the home care model to the social model of retirement is beneficial for the elderly care system as a whole. Among the factors mentioned above, sense of access, satisfaction, and security reflect the mental health issues that widely affect elderly people, and they can be unified under the concept of “subjective wellbeing.” Specifically, an elderly person's subjective wellbeing is an overall assessment and comprehensive psychological index of their quality of life, according to their own criteria (22). Concern for the subjective wellbeing of elderly people is a major practical issue, which reflects the ultimate policy goal of improving people's wellbeing and has far-reaching practical significance behind it (23–25). Social pension level is also an important factor affecting subjective wellbeing among elderly people (26); therefore, subjective wellbeing functions as an intermediary or bridge between social pension level and transformation of the elderly care model. Overall, pension levels and subjective wellbeing among elderly people are closely related. Both are influential factors in the choice of elderly care model, and the mechanisms by which these influences operate are complex. Paying attention to these mechanisms is important in making improvements to pension insurance policy, constructing social pension services, enhancing subjective wellbeing among elderly people, and improving the system for provision of social pension services.

Based on data from the 2018 China Longitudinal Aging Social Survey (CLASS), the independent variable of pension level among the elderly population is examined in this study in terms of its influence on the dependent variable of preferences regarding different models of elderly care, such as home, community, or institutional care. Subjective wellbeing is treated as a mediating variable. In terms of methods, a structural equation model is constructed for empirical testing, path analysis, and decomposition of effects. The empirical results are also examined for heterogeneity between sub-groups of the sample in terms of age, household registration status, education level, number of children, and gender. The results show that increasing pension level can significantly improve subjective wellbeing among elderly people. Increasing an individual's pension level also reduces their preference for home-based care and promotes the choice of community or institutional care. In addition, the paths by which these factors influence elderly people's preferences vary by gender, age, household registration, marital status, health status, education level, number of children, and gender of children.

The novel contributions of this paper are as follows. First, in terms of topic selection, this paper discusses the influence of pension levels on mental health and choice of care model among elderly people, with their willingness to choose particular models of care treated as explained variables and subjective wellbeing as a mediating variable. The study explores this unique and complex impact path, taking into account existing research on physical and mental health in the elderly population. The paper therefore provides a theoretical basis for the formulation of China's pension policy and the construction of elderly care institutions. It also makes a contribution to the improvement of provision of elderly care services and of people's wellbeing. Second, in terms of research methods, data on various dimensions are used in this study to construct latent variables representing pension level, subjective wellbeing, and preferences regarding model of elderly care among elderly people. In addition, a structural equation model is built in accordance with the proposed hypotheses and the potential pairwise relationships between different elements are analyzed. It is difficult to analyze and measure a very large number of variables using a multiple regression model. In contrast, structural equation modeling has the advantage of overcoming biased endogeneity estimates that have arisen in previous studies using multiple regression. Third, in terms of research content, not only does this article outline the influence paths and decompose the direct and indirect effects of pension level and subjective wellbeing on individuals' preferences with respect to three models of elderly care under the same framework, but it also presents the findings (based on heterogeneity analysis, which requires the application of different analyses for different sub-groups of research participants) that the mechanism of influence varies across several sub-groups of elderly people, according to factors such as gender, age, household registration status, marital status, and number of children. This study thus bridges the gap in the research field pertaining to China's retirement industry and provides a practical and effective reference for the promotion of a socialized retirement process. It constitutes useful reference material for the promotion of socialized aging.

Following this introductory section, the theoretical analysis framework is outlined in Section 2, including a literature review and discussion of the research hypotheses to be tested. The data source, selection of variables, and statistical methodology are described in Section 3, and the results are presented in Section 4. In Section 5, these results are discussed and conclusions are presented.

## 2. Theoretical analysis framework

### 2.1. Literature review

The strong trend toward aging populations has prompted close attention to the elderly population. In addition to the themes of physical health, economic status, and participation in the community (6, 27, 28), subjective wellbeing among elderly people and the practical subject of elderly care services are also current topics in this field.

In the field of psychology, *subjective* wellbeing refers to people's overall emotional and cognitive judgment of their current quality of life; it is comprised of emotional experience and life satisfaction, and is characterized by subjectivity, stability, and wholeness (29, 30). Measures of subjective wellbeing make use of methods including surveys, interviews, and experimental methods; currently, scale measures are most widely used. For instance, Zhang et al. (31) updated the Social Production Function Instrument to measure five dimensions of subjective wellbeing, including emotion, behavioral confirmation, status, comfort, and motivation. The instrument passed an assessment of its reliability and validity when applied in a community of elderly individuals. Additionally, Chen (32) created a subjective wellbeing scale with acceptable reliability and validity based on three dimensions: self-completeness, life satisfaction, and family harmony.

Most studies on the topic have indicated that participation in social insurance can improve people's subjective wellbeing by assisting them in reducing risk (33–37). In contrast, unfairness in social insurance schemes can have the opposite effect (34, 35). In the specific domain of social pension insurance, the correlation between social pension income and subjective wellbeing among elderly people is controversial. Liu (36) conducted an empirical study of the effect of social security systems on happiness. The results showed that the provision of both medical insurance and pensions has a significant positive relationship with subjective wellbeing, and the impact is even more significant in the case of rural residents and low-income individuals. Deng and Yang (37) focused on the effects of pension income and individual consumption on the subjective wellbeing of rural elderly people in China. The results show that endowment insurance has a positive role in promoting the subjective well being of rural elderly in China, and it can also alleviate the negative impact of consumption differences on the subjective well being of rural elderly in China. This forms a contrast with the findings of Yue and You (38), who used an ordered probit model analysis of data from a survey on public welfare attitudes in Guangdong Province to examine the relationship between people's perceptions of pension contributions and their subjective wellbeing. According to this research, a high level of subjective wellbeing is often accompanied by a low degree of pension awareness, and participation in a social pension scheme does not efficiently improve wellbeing among middle- and low-income groups. Hence, it is necessary to reflect on whether social pension policies can genuinely improve the subjective wellbeing of individuals and contribute to the improvement of pension policies.

In addition to social insurance, there are many other factors influencing subjective wellbeing, including social factors, family factors, and individual psychological factors. First, elderly people's subjective wellbeing is influenced by geography, with variation in levels of subjective wellbeing observed between coastal and inland areas, and between urban and rural areas. Subjective wellbeing among elderly people living in rural areas is closely related to the policy system and their financial status, while relationships with offspring are more significant among elderly people living in urban areas (39–41). Due to the absence of their children's help and company, elderly individuals who live alone frequently experience loneliness and report poorer subjective wellbeing (42), but support and aid from their children can enhance older people's subjective wellbeing (43, 44). In addition, elderly people who participate in educational activities can achieve higher subjective wellbeing through a good educational atmosphere and experience (45). Elderly people who use social media more often experience positive emotions, and their subjective wellbeing is promoted (46). Through the use of an ordered logit model and Shapley value disaggregation, Wang and Zhao (47) discovered that factors including living arrangement, household registration status, income level, age, and health insurance participation have significant effects on the subjective wellbeing of older adults.

In terms of elderly care models, the main care model in China has long been the family care model, which relies on an individual's children to provide economic support, spiritual comfort, and daily care; the aging population trend makes this traditional model unsustainable. In the course of history, the introduction of pension plans covering low-income groups has been observed to disrupt the traditional family care model to varying degrees and promote the development of social care; examples include Taiwan's “Farmers' Insurance,” Mexico's “Elderly Nutrition Plan,” and South Africa's “Elderly Pension Plan” (19). Since the implementation of the New Rural Social Pension Insurance in China in 2009, many scholars have explored whether this scheme has altered Chinese residents' views on different models of elderly care. Several scholars have evaluated the impact of China's New Rural Social Pension Insurance on rural residents' choices regarding model of elderly care; they have found that the new insurance system can improve the economic and life independence of the elderly population, and to some extent, it has altered China's traditional model of elderly care provision by children, specifically by introducing the possibility of elderly people living separately from their children (19). However, this “social care model” has a limited role in replacing the “family care model.” Further improving pension levels is conducive to improving the “social care model” in rural China (20). Hao et al. (21) have replicated these findings on the effect of the New Rural Social Pension Insurance system, drawing a conclusion that differs from those of previous publications in the literature: that is, “family intergenerational care” has significantly squeezed out “individual self-care,” and the latter has no significant impact on the former. Mu (48) further proposes that the substitution rate of “care by children” and “personal care” is related to the number of children, and the marginal substitution rate decreases as the number of children increases. In addition, individuals with only one child and parents participating in the social pension insurance scheme are more willing to save for a personal pension. The social care model has a crowding effect on personal pension savings, because it replaces some functions of the family care model (49).

At present, there are few studies reported in the literature on the impact of elderly people's subjective wellbeing on their choice of pension mode, but most studies show that there is a correlation between the two. Specifically, elderly people who choose different care models will have different perceptions in relation to social support and loneliness, resulting in differences in subjective wellbeing. Related studies have shown that those who choose home-based elderly care receive the highest levels of social support and experience lower levels of loneliness. Therefore, the subjective wellbeing of older adults who choose home-based care is usually higher than that of those who receive care in the community or in institutions (50). Chen and Zhang (51) used ordered probit models and SEM to examine the effects of different elderly care models on subjective wellbeing among rural elderly people in China; similarly, they found that home-based care remains an important factor in enhancing the wellbeing of rural elderly people. Qu and Zhang (52) conducted a study to analyze the subjective wellbeing of elderly people with different attitudes toward elderly care in China, taking five cities in Shandong Province as examples. The findings of this research were that a model combining home-based care and community care is more in line with elderly people's wishes and is more conducive to improving their subjective wellbeing, while the institutional model of elderly care is acceptable only in specific scenarios. Thus, there are significant differences in subjective wellbeing between elderly people who choose different elderly care models. Potential explanations for this phenomenon are differences in social traditions, constraints imposed by the current level of economic development, and imperfections in service infrastructure. Moreover, an elderly individual's choice of care model is affected by such factors as their individual characteristics, family status, economic status, and pension risk awareness (53).

As indicated above, pension insurance participation is an important factor influencing the subjective wellbeing of older people and their choice of elderly care model, and differences in the perceived subjective wellbeing of older people are closely related to their choice of old-age security scheme. Currently, findings on the effects of China's pension policy on subjective wellbeing and choices of care model among elderly people are still controversial. Few scholars have examined the influence of elderly people's intentions of participating in social pension insurance schemes and their pension income level on their specific preferences for home-based, community-based, or institutional models of care.

Therefore, in this study, further research and analyses were conducted to investigate the mechanisms and paths underlying the relationships between social pension participation, subjective wellbeing, and elderly care choices. This paper aims to fill the research gaps in this field and lay the theoretical foundation for subsequent development.

### 2.2. Research hypotheses

Based on the analysis above, it can be seen that pension income level affects the subjective wellbeing of elderly people, and that the level of pension benefits and subjective wellbeing among elderly people in China are the two major factors affecting their choice of care model. In general, social pension benefits can help older people to mitigate potential risks and can increase their income in their old age, thus altering their willingness to choose certain care models by improving their subjective wellbeing; this indicates that the subjective wellbeing could potentially mediate the effect of social pension level on choice of elderly care model.

Accordingly, the following research hypotheses are proposed:

Hypothesis 1: An increase in pension level has a positive effect on subjective wellbeing among elderly people.

Hypothesis 2: Pension level affects choice of elderly care model.

Hypothesis 3: The subjective wellbeing of elderly people mediates the influence of their pension level on their choice of elderly care model.

In accordance with the available survey data, pension level was measured on the basis of the practical situation of each respondent. The specific indicators were: the logarithm of their monthly social pension income; the ratio of this monthly amount to their monthly expenditure; and the ratio of this monthly amount to their total monthly income.

Subjective wellbeing was measured on three dimensions (life satisfaction, emotional pleasure, and social harmony), and all three of these were used as indicator variables.

Elderly people's perceptions of home-based care were measured on the basis of several variables: their willingness to opt for home-based care; their desire to live in their children's home; their expectations of care being provided by their children; and their degree of emotional attachment to their children.

Similarly, elderly people's perceptions of community care were measured on the basis of their willingness to opt for community care, their willingness to purchase and previous usage of community services, and their satisfaction with the community environment.

Finally, the variables indexing elderly people's perceptions of institutional care were their willingness to opt for institutional care, their family members' willingness, and their understanding and impression of nursing homes.

**Figure 1:**
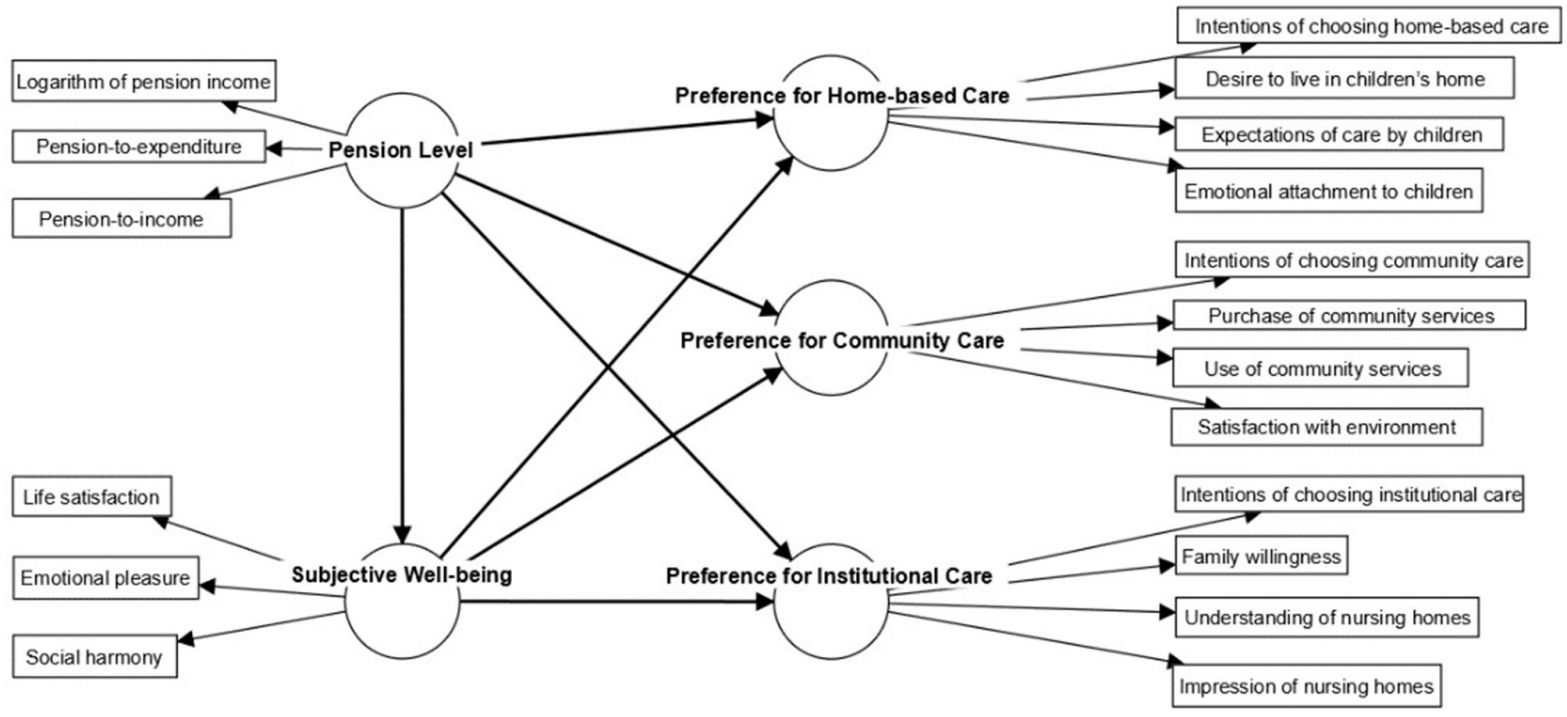
Research framework.

In summary, a complete research framework covering social pension level, choice of elderly care model, and subjective wellbeing was established for the purpose of this study, as illustrated in Figure 1.

## 3. Data and methodology

### 3.1. Data source

This study primarily used questionnaire data from the 2018 China Longitudinal Aging Social Survey (CLASS), which is a continuous, large-scale nationwide social survey project. CLASS launched in 2014 as the first nationwide baseline survey and has been followed up every 2 years since then. It regularly and systematically collects socio-economic data on older people in China in order to understand the difficulties and challenges they face. CLASS aims to evaluate the effectiveness of social policies and improve the quality of life of older people, providing an important theoretical foundation and factual basis for a proactive elderly security policy in China. Using data from CLASS is time-efficient and practical, and the data are nationally representative. The dataset contains measures of all the variables involved in this study, namely, pension level, choice of elderly care model, and subjective wellbeing among elderly people.

### 3.2. Selection of variables

As shown in Figure 1, the set of latent variables in this study consisted of pension level, subjective wellbeing, and preferences for home-based care, community care, and institutional care. Each of these latent variables was measured *via* a series of indicator variables, as laid out above. Table 1 presents the latent variables, the associated indicator variables, and the grouping variables selected for the heterogeneity analysis conducted in this study. The specific questions used to measure the indicator variables and the form of their assigned values are also provided in this table.

**Table 1:** Selection and measurement of variables.

**Latent variables**	**Indicator variables**	**Code**	**Questions**	**Form of variable values**
Pension level (PL)	Logarithm of pension income	PL1	How much pension income do you receive monthly? (Record specific number, including basic pension for enterprise employees, pension for public institutions, and basic pension for urban and rural residents)	ln (total pension income amount +1)
	Pension-to-expenditure ratio	PL2	How much pension income do you receive monthly? (As above)	Pension income/monthly per capita household expenditure
			What was the average total monthly expenditure for your family (family members who eat and live together) in the past 12 months? (Record specific numbers)	
	Pension-to-income ratio	PL3	How much pension income do you receive monthly? (As above)	Pension income/monthly per capita household income
			What was the average total monthly income for your family (family members who eat and live together) in the past 12 months? (Record specific numbers)	
Subjective wellbeing (SW)	Life satisfaction	SW1	Do you think the following descriptions match your current situation? (Willing to participate in neighborhood council activities; interested in doing something for society; enjoy learning, feel useful to society; have difficulty fitting in with social changes; have difficulty accepting growing views; have difficulty accepting increasing number of new social policies; feel that social changes are becoming increasingly unfriendly to the elderly)	Not at all = 0; Rarely match = 1; Fairly match = 2; Relatively match = 3; Totally match = 4 (Responses to all 7 items are summed; some items are reverse-scored based on their content)
	Emotional pleasure	SW2	Next, I would like to know how often you have these feelings in the last week. (Do you feel good? Are you lonely? Are you sad? How is your life? How is your appetite? How is your sleep? Are you doubtful about your own capabilities? Do you feel like you have nothing to occupy yourself with? Do you have a lot of fun in life? Do you feel you have no one to keep you company? Do you feel you are ignored or isolated by others?)	Never = 0; Sometimes = 1; Often = 2 (Responses to all 12 items are summed; some items are reverse-scored based on their content)
	Social harmony	SW3	Social support and social connections. (How many family members and relatives/friends can you meet or contact at least once a month? How many family members and relatives/friends can you talk with about your personal business in confidence? How many family members and relatives/friends do you have who can help you when you are in need?)	No one = 0; Only one = 1; 2 people = 2; 3-4 people = 3; 5-8 people = 5; 9 or more = 9 (scores for all 6 items are summed)
Preference for home-based care (HC)	Intentions of choosing home-based care	HC1	What is your life plan in terms of elderly care?	Live in own home or offspring's home = 1; Other response = 0
	Intentions of living in children's home	HC2	Do you plan to live in your children's home when you need elderly care?	Live = 3; Uncertain = 2; Not live = 1
	Expectations of care provided by children	HC3	Do you plan to rely on your children for your elderly care needs?	Fully rely on = 3; Partially rely on = 2; Never rely on = 1 (Add together by total number of children)
	Emotional attachment to children	HC4	I will share some thoughts on filial piety and elderly care with you, and can you tell me your opinion about it: Children's emotional care for their parents is more important than financial support.	Totally agree = 5; Partially agree = 4; Not sure = 3; Partially disagree = 2; Totally disagree = 1
Preference for community care (CC)	Intentions of choosing community care	CC1	What is your life plan in terms of elderly care?	Make use of community-based elderly day-care stations or elderly nurseries = 1; Other responses = 0
	Purchase of community services	CC2	Do you think you would you pay for this service? (Home visits, hotline for seniors, escort service for medical appointments, help with daily shopping, legal assistance, home chores, senior dining hall or meal delivery, community-based day-care station or nursing home, psychological counseling)	Yes = 1; No = 0 (scores for all 9 items are summed)
	Use of community services	CC3	Have you ever used this service? (Items as above)	Yes = 1; No = 0 (scores for all 9 items are summed)
	Satisfaction with community environment	CC4	How satisfied are you with the following in your community (village or neighborhood)? (Road conditions, fitness/activity facilities, security of the environment, environmental sanitation, respect for the elderly, competence of the residents' committee staff, road and street lighting, barrier-free facilities)	Very satisfied = 5; Relatively satisfied = 4; Fairly satisfied = 3; Relatively unsatisfied = 2; Very unsatisfied = 1 (scores for all 8 items are summed)
Preference for institutional care (IC)	Intentions of choosing institutional care	IC1	What is your life plan in terms of elderly care?	Nursing home = 1; Other responses = 0
	Family willingness	IC2	Would your family want you to live in a nursing home?	Unwilling = 1; No idea = 2; Haven't reached a consensus = 3; Willing = 4
	Understanding of nursing homes	IC3	How much do you know about nursing homes?	Not familiar with them = 1; Know something about them = 2; Familiar with them = 3
	Impression of nursing homes	IC4	What is your impression of nursing homes like?	Bad = 1; Fair = 2; Good = 3
**Grouping variables**				
Gender			Male = 1, Female = 2	
Age			Number of years	
Household registration			Registered as rural = 1, Registered as non-rural = 2	
Marital status			Without spouse = 1, With spouse = 2	
Health			Very unhealthy = 1, Relatively unhealthy = 2, Fair = 3, Relatively healthy = 4, Very healthy = 5	
Education			Illiterate = 1, Sishu/literacy class = 2, Elementary school = 3, Middle school = 4, High school/junior high school = 5, College = 6, Bachelor's degree and above = 7	
Number of children			Grouped by specific number	
Number of sons			Grouped by specific number	
Number of daughters			Grouped by specific number	

### 3.3. Methodology

In this study, the primary methodology was structural equation modeling, which was used to empirically test the relationship between pension level, subjective wellbeing, and care model preferences among elderly people. This analysis was carried out using the Stata and SmartPLS software packages.

Structural equation modeling (SEM) is a multivariate statistical analysis technique that combines multiple regression and factor analysis methods for the automatic assessment of a series of interrelated causal relationships. According to the analysis presented above, a variety of subjective factors complicate the factors that determine people's preferences regarding elderly care models and their subjective wellbeing, making it difficult to directly determine the causal relationships between these variables. These factors will result in inefficient and unconvincing results if traditional methods such as multiple regression are used for data analysis. SEM is similar to multiple regression in terms of use, but is more suitable for multiple complex conditions. Therefore, in this study, structural equation modeling was applied to estimate the effects of pension level and subjective wellbeing on elderly people's preferred model of elderly care.

First, in the measurement model, the relationships between the latent variables and their indicators were assessed using SmartPLS software; this included calculation of Cronbach's alpha, Dijkstra–Henseler's rho, composite reliability, AVE, the Fornell–Larcker criterion, and the HTMT ratio for each latent variable. These results indicated that the selected indicators were appropriate: that is, there was high commonality between them, and they could be validly used to measure the corresponding latent variables (4.2.1).

The second step was to use the Stata software package to carry out structural equation modeling, constructing the preset path model between the variables as shown in Figure 1. The Maximum Likelihood (ML) estimation method was adopted and the maximum number of iterations was set at 500. The correlations between the indicator variables and latent variables were re-verified in the measurement model (4.2.1); the size, direction, and significance of the relationships between the latent variables in the structural model were computed (4.2.2); and the research hypotheses were tested. Finally, the impact paths were decomposed and the direction and proportion of direct and indirect impact paths were explored (4.3).

The third step, for the heterogeneity analysis, was to divide the sample into groups according to each of the grouping variables, and repeat the analyses described in the second step to identify differences in the influence of each variable and the path of influence in different sub-groups (4.4).

## 4. Results

### 4.1. Descriptive analysis

The survey from which the data analyzed in this study were drawn focused on the elderly population (people over 60 years old) in China. A total of 4,422 valid sets of responses from the database were retained after missing and invalid values were eliminated *via* filtering and sorting. Of these respondents, 3,645 were participating in a social pension scheme, accounting for 82.43% of the total sample. Table 2 provides detailed descriptive statistics on each of the categories of variables analyzed in this study. It can be seen that, across the entire sample, the logarithm of the monthly pension amount received had a minimum value of 0 and a maximum of 10.3086, and the mean value was 6.0897. These values correspond to the specific amounts of 0 yuan (minimum), 29,988 yuan (maximum), and 4,631.0810 yuan (mean), while the median was only 1,800 yuan, which shows that the distribution of pension income in China is remarkably polarized. The minimum ratio of monthly pension to per capita household expenditure was 0, the maximum was 9.9960, the median was 0.3333, and the average was 1.8083. The minimum ratio of monthly pension to per capita household income was 0, the maximum was 11.1067, the median was 0.05038, and the average was 0.1887. These figures reveal that most elderly people would not be able to meet their daily needs if they relied solely on their pension income. In contrast, a small number of respondents had excess or surplus pension. Therefore, the overall distribution and fairness of pensions for elderly people in China needs to be improved. In addition, a comparison of social pension scheme participants and non-participants showed that participants in such a scheme experienced a slightly higher degree of emotional pleasure and perceived social harmony, and they were more likely to recognize the value of community and institutional care models, were more willing to purchase community services, and generally had higher levels of education. These findings preliminarily support the hypothesis of this study, but the complex mechanisms underlying these relationships still needed to be rigorously and empirically tested.

**Table 2:** Descriptive statistics.

**Variables**		**Entire sample (*****N*** = **4,422)**					**Participating in social pension scheme (*****N*** = **3,645)**		**Not participating (*****N*** = **777)**	
		**Mean**	**Standard error**	**Min**	**Max**	**Median**	**Mean**	**Standard error**	**Mean**	**Standard error**
Pension level	Logarithm of pension income	6.0897	3.4240	0	10.3086	7.4961	7.3879	2.1518	0	0
	Pension-to-expenditure ratio	1.8083	3.4631	0	10.5221	0.3750	2.1938	3.7020	0	0
	Pension-to-income ratio	0.1887	0.4606	0	11.1067	0.0538	0.2289	0.4982	0	0
Subjective wellbeing	Life satisfaction	24.4844	4.1743	8	40	24	24.5816	4.1283	24.0283	4.3130
	Emotional pleasure	28.1156	4.0220	13	36	28	28.2131	4.0485	27.6577	3.8651
	Social harmony	15.2906	7.3169	0	54	14	15.8458	7.3216	12.6860	6.7116
Preference for home-based care	Preference for home-based care	0.9297	0.2557	0	1	1	0.9215	0.2689	0.9678	0.1766
	Intentions of living in children's home	4.4466	2.6101	0	15	4	4.3745	2.5851	4.7851	2.7005
	Expectations of care being provided by children	5.2151	2.7828	0	15	5	5.0508	2.7132	5.9858	2.9713
	Emotional attachment to children	4.1228	0.8127	1	5	4	4.1534	0.8009	3.9794	0.8523
Preference for community care	Preference for community care	0.0267	0.1612	0	1	0	0.0302	1.1711	0.0103	0.1010
	Willingness to purchase community services	0.4959	1.3245	0	9	0	0.5684	1.4080	0.1557	0.7376
	Use of community services	0.2146	0.7404	0	9	0	0.2145	0.7394	0.2149	0.7454
	Satisfaction with community environment	29.6861	4.8716	8	40	30	29.8082	4.8160	29.1133	5.0889
Preference for institutional care	Preference for institutional care	0.0436	0.2043	0	1	0	0.0483	0.2144	0.0219	0.1464
	Family willingness	1.7854	0.9838	1	4	1	1.7893	0.9981	1.7671	0.9144
	Understanding of nursing homes	1.8643	0.6574	1	3	2	1.8875	0.6534	1.7555	0.6654
	Impression of nursing homes	1.9808	0.5734	1	3	2	1.9805	0.5742	1.9820	0.5696
Grouping variables	Gender	1.4765	0.4995	1	2	1	1.4815	0.4997	1.4530	0.4981
	Age	71.0821	7.1447	60	100	69	70.9268	7.1408	71.8108	7.1224
	Household registration	1.5149	0.4998	1	2	2	1.5720	0.4949	1.2471	0.4316
	Marital status	1.7069	0.4552	1	2	2	1.7204	0.4488	1.6435	0.4793
	Health	3.4358	0.8475	1	5	4	3.4519	0.8370	3.3604	0.8918
	Education	3.0620	1.3587	1	7	3	3.1720	1.3431	2.5457	1.3124
	Number of children	2.4765	1.3543	0	6	2	2.4310	1.3419	2.6898	1.3923
	Number of sons	1.2953	0.9378	0	6	1	1.2667	0.9369	1.4299	0.9309
	Number of daughters	1.1811	1.0717	0	7	1	1.1643	1.0683	1.2580	1.0850

### 4.2. Structural equation modeling

**Figure 2:**
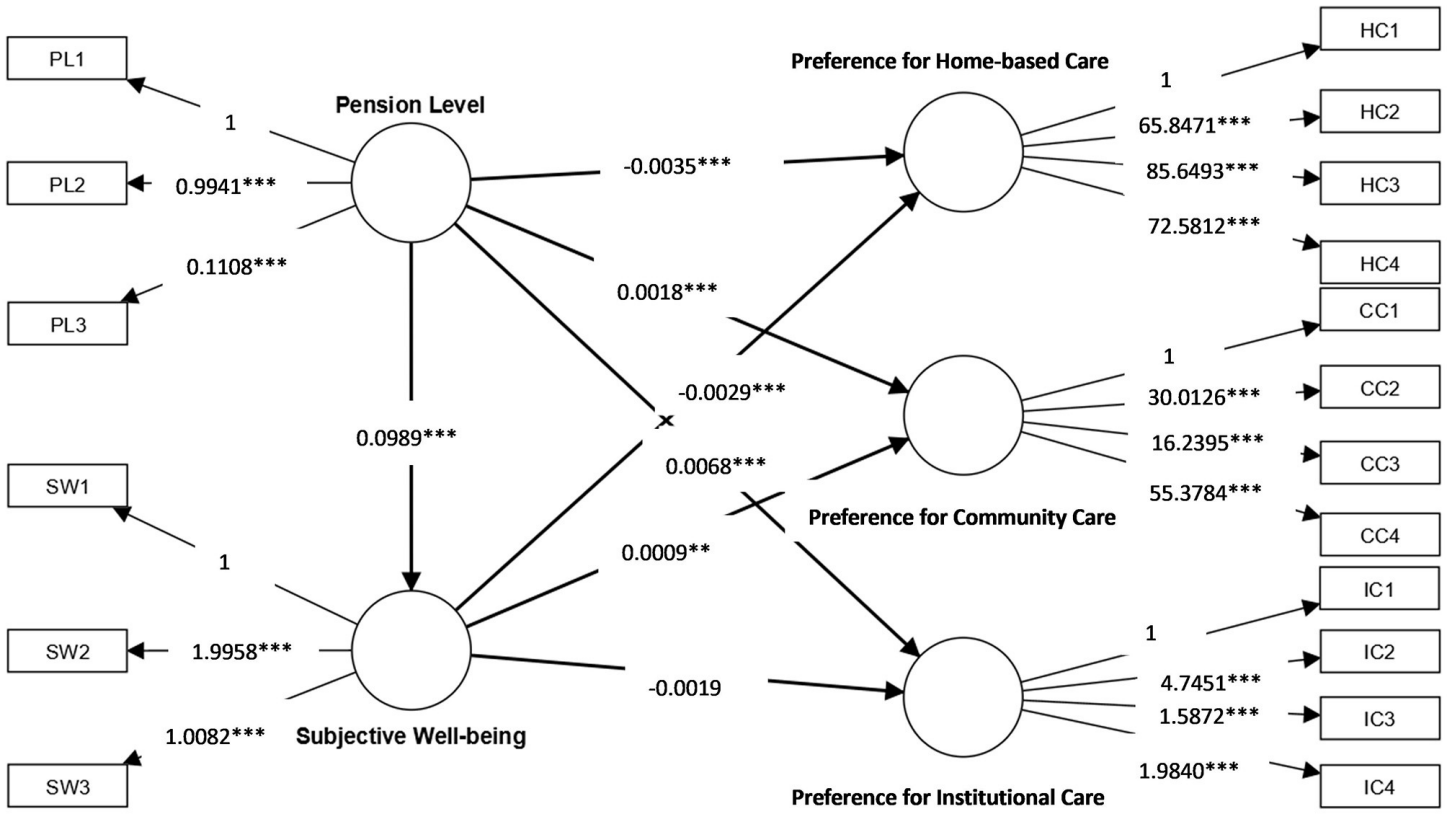
Model results. ^***^, ^**^, and ^*^ indicate significance at the 1, 5, and 10% levels.

Figure 2 and Table 3 show the estimated results of both the measurement model and the structural model for the effects of social pension level and subjective wellbeing on elderly people's elderly care model preferences; these results test the research hypotheses listed in Section 2.

**Table 3:** Baseline regression results.

**Model**	**Relationships between variables**		**Coefficient**
Structural model	PL → SW		0.0989^***^
	PL → HC		−0.0035^***^
	SW → HC		−0.0029^***^
	PL → CC		0.0018^***^
	SW → CC		0.0009^**^
	PL → IC		0.0068^***^
	SW → IC		−0.0019
Measurement model	PL	PL1	1
		PL2	0.9941^***^
		PL3	0.1108^***^
	SW	SW1	1
		SW2	1.9958^***^
		SW3	1.0082^***^
	HC	HC1	1
		HC2	65.8471^***^
		HC3	85.6493^***^
		HC4	72.5812^***^
	CC	CC1	1
		CC2	30.0126^***^
		CC3	16.2395^***^
		CC4	55.3784^***^
	IC	IC1	1
		IC2	4.7451^***^
		IC3	1.5872^***^
		IC4	1.9840^***^
Fit indices	*R*^2^ = 0.6857; CFI = 0.8870; TLI = 0.8410; RMSEA = 0.0800; SRMR = 0.0630; CD = 0.6860	

The factor loading coefficients of life satisfaction, logarithm of pension income, preference for home-based care, preference for community care, and preference for institutional care were automatically set to 1. ^***^, ^**^, and indicate significance at the 1% and 5% levels, respectively.

#### 4.2.1. Measurement model

A measurement model uses indicator variables to measure latent variables, thereby characterizing the relationship between the latent and indicator variables. In this study, the latent variables mentioned above (pension level, subjective wellbeing, and preferences for home-based care, community care, and institutional care) were all reflective ones, and the direction of causal influence was from the latent variable to each indicator variable. Thus, all five latent variables were entered into a Reflective Measurement Model (RMM).

Taking the reflective latent variable “pension level” as an example, the measurement model is shown in Equation (1).


(1) $$\begin{array}{l} Y\;=\;\Lambda_{y}\eta +\varepsilon \end{array}$$

Equation (1) represents a factor η, in this case pension level, measured by three indicator vectors Y: log of pension income *y*_1_, pension-to-expenditure ratio *y*_2_, and pension-to-income ratio *y*_3_. Λ_*y*_ is the factor loading coefficient between each measured indicator variable and the explained latent variable. Finally, ε is the measurement error of the explanatory latent variable.

Table 4 presents the results for each of the latent variables considered in the structural equation model. Given that Cronbach's alpha, Dijkstra–Henseler's rho, and the composite reliability statistic were above 0.7 in almost all cases, the reliability of the latent variables was confirmed (54). The average variance extracted (AVE) exceeded 0.5, supporting the convergent validity of the model (55). Since the square root of each latent variable's AVE was greater than the correlation of that variable with the other latent variables, the Fornell–Larcker criterion supported the discriminant validity of the model (56). Additionally, the heterotrait–monotrait ratios (HTMT values), which were all below 0.85, showed that each latent variable was a valid discriminant construct (57).

**Table 4:** Measurement model evaluation.

	**Pension level**	**Subjective wellbeing**	**Preference for home-based care**	**Preference for community care**	**Preference for institutional care**
**Construct reliability and average variance extracted**					
Cronbach's alpha	0.8650	0.6920	0.7280	0.7000	0.7110
Dijkstra–Henseler's rho	0.9610	0.6950	0.7340	0.7140	0.7210
Composite reliability	0.7770	0.7003	0.7250	0.7100	0.7150
AVE	0.6520	0.5560	0.6240	0.5780	0.6110
**Fornell–Larcker criterion**					
PL	0.8070				
SW	0.1580	0.7460			
HC	−0.2200	−0.2660	0.7900		
CC	0.1940	0.0800	0.1900	0.7600	
IC	0.1350	−0.0950	0.1590	0.1970	0.7820
**HTMT**					
PL					
SW	0.2260				
HC	0.3120	0.5180			
CC	0.2530	0.4110	0.7940		
IC	0.1670	0.2670	0.7390	0.4930	

Similarly, as shown in Table 3, when the factor loading coefficients of life satisfaction, logarithm of pension, and preference for home-based care were set to 1, the estimated coefficients between each other indicator variables and the corresponding latent variable in each of the three measurement models were all positive and significant at the 1% level; this again verifies that the selected indicator variables could function as efficient measures of each latent variable.

#### 4.2.2. Structural model

A structural model describes the relationship between the explanatory and explained latent variables. The relationships between variables in the path model constructed in this study were intricate. For example, the portion of the structural model aiming to characterize the relationship between pension level, subjective wellbeing, and preference for home-based care was:


(2) $$\begin{array}{l} \eta \;=\;\gamma_{1}\xi_{1}+\gamma_{2}\xi_{2}+\zeta \end{array}$$

In Equation (2), η is the explanatory latent variable “home-based care choice.” The latent variable ξ_1_, representing pension level, is measured by three indicator variables (log of pension income, pension-to-expenditure ratio, and pension-to-income ratio), and the latent variable ξ_2_, subjective wellbeing, is also measured by three indicators (life satisfaction, emotional pleasure, and social harmony). The coefficients representing the influence of pension level and subjective wellbeing on preference for home-based care are γ_1_ *and γ*_2_, respectively; ζ represents the prediction error in each case. The estimation method used to estimate the influence coefficients in this study was the maximum likelihood (ML) method, which is widely used and the most accurate method available at present.

As shown in Figure 2 and Table 3, in the regression results for preference for home-based care, the regression coefficient representing the effect of the latent variable “pension level” on this outcome was negative and significant at the 1% level, with a magnitude of −0.0035. This indicates that the higher the level of social pension benefits an elderly person has, the less likely they will be to choose home-based care. Specifically, for every 1-unit increase in pension level, their preference for home-based care decreases by 0.35%. Meanwhile, the coefficient for the latent variable of subjective wellbeing was also negative and significant at the 1% level, with a magnitude of −0.0029, indicating that the higher an elderly person's subjective wellbeing, the less likely they will be to choose home-based care. For every 1-unit increase in pension level, their preference for home-based care decreases by 0.29%. Additionally, according to the regression results for subjective wellbeing, the regression coefficient representing the effect of pension level on subjective wellbeing was positive and significant at the 1% level, with a magnitude of 0.0989, which indicates that increasing elderly people's social pension level can improve their subjective wellbeing. For every 1-unit increase in pension level, their subjective wellbeing increases by 9.89%. On the basis of these findings, this study indicates that elderly people's social pension level can exert a direct and negative influence on their preference for home-based care, and that this influence is achieved in part *via* an increase in their subjective wellbeing.

In terms of the regression results for preference for community care, the regression coefficient representing the effect of the latent variable of pension level on this outcome was positive and significant at the 1% level, with a magnitude of 0.0018, which shows that the likelihood of preferring community care among elderly people increases with an increase in social pension level. For every 1-unit increase in the level of pension benefits, their preference for community care increases by 0.18%. The coefficient for the latent variable of subjective wellbeing was also positive and was significant at the 5% level, with a magnitude of 0.0009, indicating that the higher the subjective wellbeing of an elderly person, the stronger their preference for community care will be: specifically, for every 1-unit increase in subjective wellbeing, their preference for community care increases by 0.09%. In addition, an increase in social pension level can enhance subjective wellbeing among elderly people. Therefore, both social pension level and subjective wellbeing have direct positive effects on their preference for community care, and improvements in social pension level increase their preference for community care *via* the mediating effect of an increase in subjective wellbeing.

In the regression results for preference for institutional care, the regression coefficient representing the effect of the latent variable of pension level on preference for institutional care was positive and significant at the 1% level, with a magnitude of 0.0068, which indicates that the higher the level of social pension benefits among elderly people, the higher their likelihood of preferring institutional care. Specifically, for every 1-unit increase in their pension level, their preference for institutional care increases by 0.68%. However, the estimated coefficient representing the influence of the latent variable of subjective wellbeing on preference for institutional care was not significant, with a magnitude of −0.0019, although pension level was found to have a positive and significant impact on subjective wellbeing among elderly people. In summary, elderly people's social pension level can exert a direct and positive influence on their preference for institutional care, but it does not exert an indirect influence *via* subjective wellbeing.

### 4.3. Decomposition of effects

The results of the basic form of structural equation modeling revealed an initial picture of the mutual influence paths and effects of each variable and confirmed the hypothesis of this study. Following this analysis, an effect decomposition analysis was conducted for each of the three influencing effects to further examine the specific influence paths and effects in operation between pension level, subjective wellbeing, and elderly care model preferences. The results are shown in Table 5.

**Table 5:** Decomposition of effects.

**Explained variable**	**Explanatory/mediating variables**	**Direct effect**	**Indirect effect**	**Total effect**	**Proportion of indirect effect**
Preference for home-based care	Pension level	−0.0035^***^	−0.0003^***^	−0.0038^***^	7.89%
	Subjective wellbeing	−0.0029^***^			
Preference for community care	Pension level	0.0018^***^	0.0001^*^	0.0019^***^	5.56%
	Subjective wellbeing	0.0009^**^			
Preference for institutional care	Pension level	0.0068^***^	−0.0002	0.0066^***^	2.86%
	Subjective wellbeing	−0.0019			

^***^, ^**^, and ^*^ indicate significance at the 1, 5, and 10% levels.

Taking preference for home-based care as the explained variable, social pension level was found to exert a significant negative direct effect on this. Meanwhile, the same variable also exerted a significant negative indirect effect on it *via* the mediating effect of subjective wellbeing. Thus, the total effect (also significant and negative) was the sum of these two effects, with the indirect effect accounting for 7.89% of the total effect.

In contrast, taking preference for community care as the explained variable, level of pension benefits was found to exert a significant positive direct effect on this variable. Pension level also exerted a significant positive indirect effect *via* the mediating effect of subjective wellbeing. Therefore, the total effect was also positive and significant, with the indirect effect accounting for 5.56% of this total effect.

Finally, taking preference for institutional care as the explained variable, social pension level exerted a significant direct positive effect on this variable, but the indirect effect *via* subjective wellbeing as a mediating variable was not significant.

In conclusion, although social pension level exerts different influences on preferences for home-based and community care among elderly people, this influence relies more on the direct effect in both cases. The indirect path plays only a supplementary role. Meanwhile, there is no indirect influence of this variable on preference for institutional care *via* subjective wellbeing.

### 4.4. Heterogeneity analysis

In this study, differences among elderly people in terms of gender, age, household registration, marital status, health status, education level, number of children, and gender of children were taken into consideration. These differences may cause significant differences in pension levels, subjective wellbeing, and elderly care model preferences. Therefore, the sample was divided into different groups for a heterogeneity analysis on various dimensions. To examine gender differences, respondents were divided into a sub-group of men and a sub-group of women. To examine age differences, 80 years of age was treated as a boundary, with elderly people older than 80 years classified as senior elderly people and others as middle-old and younger. In terms of household registration status, the sub-groups were rural elderly people and non-rural elderly people. The sample was also divided according to marital status (with spouse vs. without spouse), health status (unhealthy vs. healthy), and education level (illiterate vs. literate). In terms of number of children, respondents were classified into three groups: those with zero children, those with one child, and those with more than one child. Finally, the sample was also divided into a group with no sons vs. a group with sons, and a group with no daughters vs. a group with daughters. In the case of all these sub-groups, regression results indicated that an increase in pension level had a significant positive effect on subjective wellbeing. However, there were differences in the effects of pension level and subjective wellbeing on elderly care model preferences among different groups of elderly people, as shown in Table 6. These findings are summarized below.

1. There was no significant difference between the regression results for the sub-group of elderly women and the overall sample. In the case of the sub-group of men, the regression coefficient representing the effect of subjective wellbeing on preference for community care was not significant. Therefore, an increase in pension level has a direct positive effect on preference for community care among elderly men, but subjective wellbeing has no mediating effect for this sub-group.
2. There was no significant difference between the regression results for the sub-group of middle-old and younger elderly people and the overall sample. In the case of senior elderly people, the regression coefficient representing the effect of pension level on preference for institutional elderly care was not significant. Additionally, the regression coefficient representing the effect of subjective wellbeing on preference for home-based elderly care was not significant, while the estimated coefficients representing its effects on preference for community and institutional elderly care were significant and negative. Therefore, subjective wellbeing cannot be regarded as a mediating variable in the relationship between pension level and elderly care model preferences among the sub-group of senior elderly people. In addition, an increase in pension level has a direct positive effect on preference for the community care model among senior elderly people, but an increase their subjective wellbeing weakens their preference for the community care model, which means that the indirect effect *via* this mediating variable is negative. Pension level does not have a significant effect on senior elderly people's preference for institutional care, and an increase in their subjective wellbeing can weaken their preference for the community care model.
3. Among elderly people with rural household registration status, the regression coefficient representing the effect of pension level on preference for institutional care was not significant. Additionally, the regression coefficient representing the effect of subjective wellbeing on preference for home-based care was not significant, and the regression coefficient representing its effect on preference for institutional care was significant and negative. Therefore, pension level does not affect rural-registered elderly people's preferences for an institutional care model. An increase in subjective wellbeing does not affect rural-registered elderly people's preferences for home-based care, but reduces their preferences for the institutional care model. In the case of urban-registered elderly people, none of the regression coefficients representing the effects of pension level on preferences for each elderly care model were significant. The regression coefficient representing the effect of subjective wellbeing on preference for community care was significant and negative. Therefore, an increase in pension level would have no impact on preferences for home, community, or institutional care among urban-registered elderly people, while subjective wellbeing is negatively related to their preference for community care.
4. For the sub-group of elderly people with spouses, there was no significant difference from the overall sample, except that the regression result representing the effect of subjective wellbeing on preference for the community care model was not significant. For the sub-group of elderly people without a spouse, the regression coefficient representing the effect of pension level on their preference for the community care model was not significant. Additionally, the regression coefficient representing the effect of subjective wellbeing on preference for home-based care was not significant, and there was a significant negative effect of this variable on their preference for community care. Therefore, pension level has no significant effect on preferences of community-based elderly care among elderly people without a spouse. Subjective wellbeing also has no significant effect on their preference for home-based care, but improved subjective wellbeing reduces their preference for community care.
5. There was no significant difference between the regression results for healthy elderly people and the overall sample. In the case of unhealthy elderly people, the regression results indicated that there was no significant effect of subjective wellbeing on their preferences for home-based or community-based elderly care. Therefore, an increase in subjective wellbeing does not produce a reduced preference for the home-based care model or an increased preference for the community care model among unhealthy elderly people.
6. Among the literate sub-group of elderly people, the only significant difference from the overall sample was that the regression coefficient representing the effect of subjective wellbeing on preference for the community care model was not significant. Among the illiterate sub-group of elderly people, the regression coefficient representing the effect of pension level on preference for the institutional model of elderly care was not significant. Additionally, the regression coefficients representing the effects of subjective wellbeing on preferences for home-based and community models of elderly care were not significant. Therefore, an increase in pension level cannot function as a factor influencing illiterate elderly people to increase their preference for the institutional model of care, and an increase in subjective wellbeing does not have an impact on their preferences for home-based or community care.
7. Among the sub-group group of elderly people with no children, neither pension level nor subjective wellbeing was found to be an influencing factor on elderly care model preferences. Among elderly people with only one child, an increase in subjective wellbeing was found to motivate a preference for the home-based care model and weaken their preference for the institutional model of care. However, pension level had no significant effect on this group's elderly care model preferences. Finally, the regression results for the sub-group of elderly people with more than one child did not differ significantly from those of the overall sample.
8. In the case of elderly people with sons, the regression results did not differ significantly from those of the overall sample. However, in the case of elderly people without sons, the regression coefficient representing the effect of pension level on preference for the institutional model of care was not significant. Additionally, the regression coefficients representing the effects of subjective wellbeing on preferences for home-based and community elderly care were not significant, and the regression coefficient representing its effect on preference for the institutional model of care was significant and negative. Therefore, the preferences of elderly people without sons for the institutional model of care are not affected by their pension level. However, their preference for the institutional model of care decreases with an increase in subjective wellbeing. In addition, their preferences for home-based or community care are not affected by their subjective wellbeing.
9. In the case of elderly people with daughters, the regression results did not differ significantly from those of the overall sample. However, in the case of elderly people without daughters, the regression coefficients representing the effects of pension level and subjective wellbeing on preferences for the community care model were not significant. Thus, neither an increase in pension level nor an increase in subjective wellbeing influences the willingness of this sub-group to choose the community model of care.

**Table 6:** Heterogeneity analysis.

**Relationships between variables**	**Gender**			**Age**		**Household registration status**	
	**Men** **(*****N*** = **2,315)**		**Women** **(*N* = 2,107)**	**Younger and middle-old elderly people** **(*N* = 3,890)**	**Senior elderly people** **(*N* = 532)**	**Rural** **(*N* = 2,145)**	**Urban** **(*N* = 2,277)**
**Preference for home-based care:**							
PL → HC	−0.0025^***^		−0.0033^***^	−0.0039^***^	−0.0027^**^	−0.0015^***^	−0.0009
SW → HC	−0.0027^***^		−0.0045^***^	−0.0027^***^	−0.0002	−0.0005	−0.0032^***^
**Preference for community care:**							
PL → CC	0.0017^**^		0.0018^**^	0.0019^***^	0.0062^*^	0.0005^*^	−0.0003
SW → CC	0.0003		0.0013^*^	0.0008^*^	−0.0350^***^	0.0007^*^	−0.0014^*^
**Preference for institutional care:**							
PL → IC	0.0048^***^		0.0089^***^	0.0079^***^	−0.0035	0.0010	−0.0011
SW → IC	−0.0029		−0.0006	−0.0005	−0.0222^**^	−0.0064^***^	−0.0013
**Relationships between variables**	**Marital status**			**Health**		**Education**	
	**Without spouse** **(*****N*** = **1,296)**		**With spouse** **(*****N*** = **3,126)**	**Healthy** **(*****N*** = **3,868)**	**Unhealthy** **(*****N*** = **554)**	**Illiterate** **(*****N*** = **994)**	**Literate** **(*****N*** = **3,428)**
**Preference for home-based care:**							
PL → HC	−0.0044^***^		−0.0024^***^	−0.0035^***^	−0.0059^**^	−0.0012^**^	−0.0037^***^
SW → HC	−0.0026		−0.0030^***^	−0.0033^***^	0.0027	−00033	−0.0030^***^
**Preference for community care:**							
PL → CC	−0.0002		0.0022^***^	0.0012^***^	0.0042^**^	0.0024^*^	0.0020^***^
SW → CC	−0.0207^***^		−0.0002	0.0009^**^	0.0014	0.0001	0.0001
**Preference for institutional care:**							
PL → IC	0.0108^***^		0.0046^***^	0.0058^***^	0.0128^**^	0.0015	0.0071^***^
SW → IC	−0.0072		−0.0007	−0.0001	0.0030	−0.0066	−0.0023
**Relationships between variables**	**Number of children**			**Number of sons**		**Number of daughters**	
	**0** **(*****N*** = **62)**	**1** **(*****N*** = **1,038)**	>**1** **(*****N*** = **3,322)**	**0** **(*****N*** = **739)**	≥**1** **(*****N*** = **3,683)**	**0** **(*****N*** = **1,268)**	≥**1** **(*****N*** = **3,154)**
**Preference for home-based care:**							
PL → HC	−0.0047	0.0001	−0.0015^***^	−0.0045^***^	−0.0022^***^	−0.0022^**^	−0.0028^***^
SW → HC	0.0127	0.0236^***^	−0.0015^***^	−0.0002	−0.0026^***^	−0.0032^***^	−0.0021^***^
**Preference for community care:**							
PL → CC	−0.0064	0.0016	0.0013^***^	0.0041^***^	0.0011^**^	0.0019	0.0014^***^
SW → CC	−0.0064	−0.0018	0.0016^***^	−0.0023	0.0013^***^	0.0009	0.0008^*^
**Preference for institutional care:**							
PL → IC	0.0216	0.0046	0.0052^***^	0.0063	0.0052^***^	0.0069^**^	0.0058^***^
SW → IC	0.0083	−0.0062^**^	−0.0021	−0.0120^**^	0.0006	−0.0010	−0.0028

^***^, ^**^, and ^*^ indicate significance at the 1, 5, and 10% levels.

## 5. Discussion and conclusions

Social and economic problems caused by the aging population are increasingly emerging in China. Both the government and families bear a heavy burden in terms of providing support with daily life for elderly people and medical care for middle-aged and elderly people, leading to a strong demand for insurance against the risks imposed by this burden.

Subjective wellbeing among elderly people is influenced by many factors. Participation in social pension security schemes can protect elderly people from the risk of shocks. In particular, access to a social pension can alleviate anxiety among the families of elderly people by subsidizing such families and enabling elderly people to maintain their dignity in their old age, which can greatly enhance quality of life and subjective wellbeing among this group. In addition, perceived emotional pleasure among elderly people is also closely related to their choice of elderly care model.

Based on existing research and perspectives on social pension levels and subjective wellbeing among elderly people, a structural equation model of the factors influencing preferences in regard to models of elderly care was constructed in this study. Data from the 2018 China Longitudinal Aging Social Survey (CLASS) questionnaire were adopted to empirically test the effects of elderly people's social pension level and subjective wellbeing on their preferences with respect to different models of elderly care. Using these data, the mediating transmission mechanisms embedded in the structural equation model were investigated, and a decomposition of effects analysis was conducted. Finally, the heterogeneity of sub-groups on multiple dimensions was also analyzed. The empirical findings were as follows.

1. An increase in social pension level significantly increases subjective wellbeing among elderly people, reduces their preference for home-based care, and encourages them to develop a preference for community and institutional care. Meanwhile, subjective wellbeing has a mediating effect on the influence of pension level on their preferences for home-based and community care.
2. The effects of pension level on preferences for home-based and community care are primarily direct effects, and the indirect effect mediated by subjective wellbeing is only supplementary. Furthermore, pension level exerts only a direct effect on preference for institutional care.
3. Heterogeneity analysis showed that there are differences in the influence paths of these effects among sub-groups of older people according to gender, age, household registration, marital status, health status, education level, number of children, and gender of children.

The conclusions of this study supplement and expand on those presented in the existing literature. First, in examining whether pension improvements promote socialization of the elderly care model, we did not constrain the scope of this study to a single scheme, namely China's New Rural Social Pension Insurance system, but included urban residents in the study and included both raw and proportional pension income as explanatory variables. The conclusions drawn here are the same as those presented in most existing publications (19–21, 48, 49), and support the view that improvements in the level of endowment insurance can indeed promote a shift among elderly people from a preference for the home-based care model to a preference for the socialized care model. Unlike previous studies, care model preferences were further divided in this study into preferences for home-based care, community care, and institutional care. We conclude that improvements in pension level weaken elderly people's preferences for home-based care, and increase their willingness to receive support in the community and in institutions, which is a new result. Additionally, and in line with most conclusions in the literature (34–38), we found that improvements in pension level can improve subjective wellbeing among elderly people, as embodied by life satisfaction, emotional pleasure, and social harmony. Finally, previous research on the relationship between subjective wellbeing and choice of care model among elderly people has focused primarily on differences in perceived wellbeing caused by the use of different care models (50–53); the general view is that elderly people cared for at home have higher levels of subjective wellbeing. In this study, pension level was treated as an explanatory variable, thereby filling a gap in the study of the impact of subjective wellbeing on choice of care model. In particular, an improvement in subjective wellbeing can weaken preferences among elderly people for home-based and institutional care, strengthening their preference for community care instead. There has been no similar research presenting a path analysis of pension level, subjective wellbeing, and choice of elderly care model under the same framework.

A limitation of this study is that, due to the limitations of the data themselves, it was not possible to divide the sample of Chinese residents into sub-groups based on the type of pension insurance scheme in which they participated in order to study the size of the impact of various types of pension insurance and differences between these effects. In addition, the respondents were not classified in sufficient detail to enable investigation of the differences between different regions. In the future, further relevant research should mitigate the above shortcomings. Such research should also further investigate the factors influencing choice of elderly care model in China, and continue to supplement and expand on the foundations of this research path, with the aim of promoting the development of socialization of the elderly care model in China and managing the aging of the population in a positive way.

## Data availability statement

Publicly available datasets were analyzed in this study. This data can be found here: http://class.ruc.edu.cn/.

## Author contributions

CS made contribution to the empirical analysis and paper composition. WL mainly focused on the theoretical analysis and paper composition. All authors contributed to the article and approved the submitted version.
